# Handmade Task Tracking Applied to Cognitive Rehabilitation

**DOI:** 10.3390/s121014214

**Published:** 2012-10-22

**Authors:** José M. Cogollor, Charmayne Hughes, Manuel Ferre, Javier Rojo, Joachim Hermsdörfer, Alan Wing, Sandra Campo

**Affiliations:** 1 Centre for Automation and Robotics CAR (UPM-CSIC), Universidad Politécnica de Madrid, José Gutiérrez Abascal 2, 28006 Madrid, Spain; E-Mails: javierrojolacal@gmail.com (J.R.); sandrabaltar@gmail.com (S.C.); 2 Institute of Movement Science, Department of Sport and Health Science, Technical University of Munich, 80992 Munich, Germany; E-Mails: charmayne.hughes@tum.de (C.H.); joachim.hermsdoerfer@tum.de (J.H.); 3 School of Psychology, The University of Birmingham, Edgbaston, Birmingham B15 2TT, UK; E-Mail: a.m.wing@bham.ac.uk

**Keywords:** apraxia, kinematics, cognitive rehabilitation, Kinect™, Activities of Daily Living

## Abstract

This article presents research focused on tracking manual tasks that are applied in cognitive rehabilitation so as to analyze the movements of patients who suffer from Apraxia and Action Disorganization Syndrome (AADS). This kind of patients find executing Activities of Daily Living (ADL) too difficult due to the loss of memory and capacity to carry out sequential tasks or the impossibility of associating different objects with their functions. This contribution is developed from the work of Universidad Politécnica de Madrid and Technical University of Munich in collaboration with The University of Birmingham. The Kinect™ for Windows^©^ device is used for this purpose. The data collected is compared to an ultrasonic motion capture system. The results indicate a moderate to strong correlation between signals. They also verify that Kinect™ is very suitable and inexpensive. Moreover, it turns out to be a motion-capture system quite easy to implement for kinematics analysis in ADL.

## 1. Introduction

The study of human movement often focuses on kinematics, describing the movements of the body through space and time, but without reference to the forces involved [1,2]. Kinematic movement analysis provides insights into the effects of maturation and development of motor learning [3], skill development [4,5], and the effects of peripheral or central nervous system injury during Activities of Daily Living (ADL) [6]. In addition, kinematic measurements can elucidate the motor strategies in goal-oriented tasks, provide essential information about a person's motor capabilities, as well as evaluate upper-extremity therapies [7].

For that reason, the study of human movements is applied to cerebrovascular diseases that affect the quality of life for individuals who suffer from such diseases. Following a stroke, up to two-thirds of patients may suffer from AADS [8]. The problem of living with AADS is that individuals are often unable to remember partial or full activities of their daily living, or execute sequential actions. So far, many projects have been developed in order to assist the rehabilitation for this kind of patients and also study the execution of manual ADL by analyzing body movements.

However, a limitation of these projects mentioned in Table 1 [9–16] is that they do not support cognitive rehabilitation. Furthermore, they typically only assist the movements or serve as a guide for the patient, teaching by repetition. Moreover, these projects do not lead to benefits in the psychological status of the patient, which is a critical factor in the rehabilitation process. For example, individuals with AADS often fail to execute everyday activities without error, and the presence of a large machine to teach the task may result in attention overload. In this situation, AADS patients and clinicians would be less likely to rely on the system for rehabilitation. Due to these limitations in current stroke rehabilitation projects, the aim of the present article is to examine the hand kinematics during the execution of an ADL for application in cognitive rehabilitation after a stroke.

Recent studies have examined the kinematics during the use of the tool in individuals with Left and Right Brain Damage (LBD and RBD) [6,17]. For example, the kinematic analysis of hammering movements has revealed that LBD patients produced smaller maximum velocity and wrist movements than healthy neurological controls [6]. In contrast, the performance of RBD patients was quite similar to that of the control subjects. Kinematic deficits were less obvious and limited to few movement parameters, in RBD (compared to LBD) patients. Strong correlations were also found between movement extension, speed parameters and the wrist movement, across the different conditions (pantomime vs. actual use), regardless of whether the values were in the normal or in the pathological range. The results of these studies indicate that the kinematic abnormalities of LBD patients during the actions from using the tool stem from a more general impairment of programming and executing goal-directed movements, and that kinematic deficits likely share a common origin.

In summary, investigating kinematic patterns of movement in naturalistic tasks such as those involved in using tools, or activities of daily living, have provided a level of insight that is not possible from standardized measurements based on ordinal scales (e.g., Fugl—Meyer assessment, nine hole peg test), and are of particular relevance to researchers and clinicians.

Several technologies have been developed in order to record body kinematics. These include cinematographic, electromagnetic (Liberty Polhemus, Flock of Birds), optoelectronic (OptoTrak, CODA, Selspot), infrared marker systems (Peak Motus, Vicon), and ultrasonic systems (Zebris). There are benefits and advantages to each kinematic analysis system. Electromagnetic systems do not suffer from marker dropouts, provide real time 6-DOF data, and are highly accurate. However, they also require that markers must be attached to body segments with cables, and the quality of data is influenced by interference from metallic objects or other magnetic fields. Optoelectronic systems employ active light emitting diodes (LED's) markers [18,19], which are triggered and pulsed sequentially using a computer. As with electromagnetic systems, the LED's must be attached to body segments via cables.

In general, the choice of each system depends on the requirements of the user but, although markers usually offer precision, popularity and the advantage of automatic tracking, which eliminates problems with marker merging or misidentication. Marker based methods have several limitations: (i) markers attached to the subject can influence the subject's movement; (ii) a controlled environment is required to acquire high–quality data; (iii) the time required for marker placement can be excessive; and (iv) the markers on the skin can move relatively to the underlying bone, leading to what is commonly called skin artefacts [20–22]. Fortunately, emerging technologies have led to the rapid development of low-cost and easy to use markerless motion capture systems, which offer an attractive solution to the problems associated with marker based methods. The most popular of these is the Kinect™, system developed by Microsoft.

In 2011, Microsoft released the Software Development Kit (SDK) focused on Kinect™, which has allowed developers and authors to develop and create different applications depending on the objective pursued with Kinect™. Developers have designed non-gaming applications for areas such as interior design (NConnex), tracking consumer behaviour (Kimetric), and for educational purposes (Kinect™ Math UWB B). Regarding the present work, the SDK has supported the corresponding functions in order to develop the correct software and interface to ensure the communication with the device and the performance of the whole system.

Considering rehabilitation applications, Kinect™, has been used before to assist in the recovery from physical and cognitive deficits after stroke [23–25]. Moreover, GestSure Technologies has developed an application to help doctors navigate MRIs and CAT scans during surgery; Jintronix has developed a software application that allows patients with recovering from stroke to perform physical therapy exercises from within their own home; and the Johns Hopkins University uses Kinect™ and gesture based tele-surgery to help in fine and precise manipulation of surgical tools while conducting surgeries.

So, in this article, a research focused on the use of Kinect™ as an adequate sensor for kinematics analysis to be applied in cognitive rehabilitation of AADS patients is presented. The main objective is to prove that Kinect™ is suitable, accurate and effective enough to be used in the analysis and movement tracking of ADL performance taking into account its low cost and easy implementation. To this end, the article is organized as follows: Section 2 describes the behaviour of AADS patients, the most common activities of daily living, and expounds upon the contribution of this article to the EU funded CogWatch project. In Section 3, more detailed information about Kinect™ is provided, including the set up and methodology used for the analysis of the data during the execution of the experiments. In Section 4, information regarding data collection and analysis of kinematic data obtained from the Kinect™ during the performance of an activity of daily living (*i.e.*, making a cup of tea) is provided. The experiment is carried out by two people: a healthy person, in order to evaluate the system more technically and a real apraxic patient, to prove the performance considering a real case of use. Moreover, Kinect™ data is then compared to the kinematic data collected at a higher sampling rate from an ultrasonic motion capture system (Zebris). The results are used to make comparison between both systems, and show the suitability of Kinect™ for investigating kinematic patterns of movement in ADL tasks. Finally, Section 5 presents the conclusions reached in the experiment.

## 2. AADS Patients and Activities of Daily Living

### 2.1. Apraxia and Action Disorganisation Syndrome Patients

A cerebrovascular disease happens when the blood supply to the brain is interrupted or there is a brain blood vessel broken. A stroke is produced due to the lack of oxygen or nutrients that the blood supplies to a specific region. Some of the symptoms could be the numbness or weakness felt in one side of the brain.

Depending on the severances of the stroke there could be multiple manifestations:

- Paralysis: weakness on a partial side of the body.
- Cognitive deficits: problems with thinking, awareness, attention, learning, judgment and memory. If severe the patient may have apraxia, agnosia or absence.
- Deficits in language: better known as aphasia.
- Emotional deficits: difficulty in controlling emotions or inappropriate emotional expressions.
- Pain: due to many factors, including damage to the sensory brain regions, inflexible joints or limb disabled.

In case of paralysis on a partial side of the body, assistive technologies have been developed to evaluate how patient is interacting with their environment, such as Brain-Computer Interfaces [26,27] or Electroculography Interfaces [28,29].

On the one hand, apraxia does not mean a movement constraint since it is defined as a neurological disorder of learned purposive movement skill that is not explained by deficits of elementary motor or sensory systems [22]. It is important to highlight that this disease not only affects the contra-lateral side of the cerebral lesion but also the ipsilateral side. For apraxia patients, it has been detected a strong correlation between the damaged side of the brain and the dysfunctional symptoms. On the other hand, Action Disorganisation Syndrome is commonly defined as a neurological disorder of cognitive errors which occur when performing familiar multiple-step tasks and which are not due to motor incapacity [30].

Although patients who suffer from AADS retain sensorimotor functions and capabilities, their cognitive ability to carry out activities of daily living is severely impaired [8]. The difficulty these patients experience in sequencing during the performance of everyday tasks means that they frequently cannot live an independent life, placing great strain on patients’ friends and families, and the local and national healthcare systems that support them [31].

AADS patients may exhibit various types of cognitive errors when performing previously familiar activities of daily living. Thus, they may omit an action (failing to add a tea bag when making a cup of tea) or using an inappropriate object (using a knife to stir the tea). Table 2 shows some of these problems and their classification.

### 2.2. CogWatch

Traditional approaches to rehabilitation for AADS include the therapist providing verbal or visual cues [32] as the patients perform selected tasks (such as making a cup of tea). Repeated practice with encouragement from the therapist, leads to improving performance and avoiding mistakes that can occur in a trial-and-error approach and at the same time avoiding the danger on the patient. It also leads to the perseveration of the mistakes.

This approach is labour intensive for therapy time and also limits the opportunity for the patient to practice the skill. For these reasons, the CogWatch project proposes a novel solution to AADS cognitive rehabilitation by providing an instrumented environment in the kitchen, including motion tracking with action recognition software, which allows cueing of the patient together with monitoring for errors.

Moreover, CogWatch turns out to be a Personal Healthcare System (PHS) that aims to deliver personalised, long-term and continuous cognitive rehabilitation of activities of daily living for stroke AADS patients at home using portable, wearable and ubiquitous interfaces and virtual reality modules. It is customized to suit the needs of individual patients at the same time as being practical and affordable for home installation so that rehabilitation takes place in familiar environments performing familiar tasks.

CogWatch system is being developed in relation to a set of scenarios involving ADL tasks. Activity of daily living comprises tasks of basic self-care such as preparing food and drinks in the kitchen, using the toilet, or washing and grooming in the bathroom. Their performance involves a sequence of component actions on environmental objects directed at some desired end goal. It is thought that successful performance depends on specifying object actions in spatiotemporal terms at a higher cognitive level and then elaborating these into specific movements of limbs, which are monitored as they progress against expected sensory consequences [33].

### 2.3. Activities of Daily Living

Studies on the performance of AADS patients in their daily living outside the lab are extremely rare. Some studies proved the disturbing effects of apraxia on daily living performance using questionnaires completed by the patients, relatives or caregivers [34]. In [35] ADL performance of stroke survivors is assessed in occupational therapy departments at general hospitals, rehabilitation centres, and nursing homes. They found a high correlation between ADL impairments and apraxia scores revealed from standard clinical tests. A coarse assessment of ADL performance at home or at the clinical ward is provided by the Functional Independence Measure (FIM). Using the FIM score as an outcome variable, a correlation between outcome and apraxia was established [36]. A comparable approach measured the amount of caregiver assistance on the Physical Self-Maintenance Scale (PSMS) and again found correlation with formal tests of apraxia [37]. Finally, a recent study in a large group of stroke patients (N = 635) noted a relatively low but consistent correlation between a multi-step action and functional independence measures such as the Barthel index or the Nottingham Extended ADL Scale [38].

An attempt to quantitatively analyse ADL performance in apraxic patients was provided in [39]. These authors measured execution time and quantified performance success during the meal received at the hospital. Analysis of the performance videos revealed that apraxic LBD patients completed less successful actions in a certain amount of time and were less efficient in organizing their meal. Interestingly, the overall time was only moderately prolonged and the prolongation was not statistically significantly. The action errors during eating correlated with severity of Apraxia as assessed by standard clinical testing.

In this case, the daily living task considered is that focused on tea making which comprises manual movements such as pouring water into the cup with a kettle, stirring a tea bag into the cup or pouring sugar with a teaspoon.

## 3. Methodology Used with Kinect™ and Set-Up for Tracking

### 3.1. Kinect™ Components

**Figure 1.**
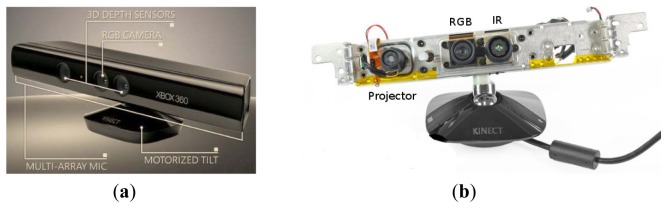
(**a**) A photo showing all the different parts of the sensor, external view of the 3D depth sensors, RGB camera, multi-array microphone, and a motorized tilt taken at Microsoft's E3 2010. (**b**) Internal view of the device from [40].

Kinect™ (Figure 1) provides a RGB camera, a depth sensor, a multi-array microphone and an infrared projector which allow:

- Full-body 3D motion capture.
- Facial recognition.
- Voice recognition.

The RGB camera is used for getting a colour image of the workplace and the infrared to obtain information about the depth of the different elements involved in the task. The method of determining 3D position for a given object or hand in the scene is described by the inventors as a triangulation process [41]. Essentially, a single infrared beam is split by refraction after exiting a carefully developed lens. This refraction creates a point cloud on an object that is then transmitted back to a receiver on the assembly.

Using complex built-in firmware, Kinect™ can determine the three-dimensional position of objects and hands in its line-of-sight by this process. The main advantage of this assembly is that it allows 3D registration without a complex set-up of multiple cameras and at a much lower cost than traditional motion labs and robotic vision apparatuses. Table 3 provides the relevant information about Kinect™ features.

### 3.2. Experimental Set Up and Software Architecture

The set-up used for the experiments is made up of the Kinect™, which is the most relevant component, and a central processor, which is responsible for: (1) receiving image and position data from the Kinect™, (2) computing all the algorithms for analysis, and (3) providing user interface to interact with the Kinect™.

It is important to mention that in order to ensure flexibility and to make the system more ergonomic and simple, the central processor and the monitor are embedded in an all-in-one computer, which provides the CPU and screen in only one device.

Finally, several objects are selected from the corresponding ADL task in order to simulate the preparation of tea. These are a kettle, a coffee cup, and a tea bag which are presented later in Figure 5.

**Figure 2.**
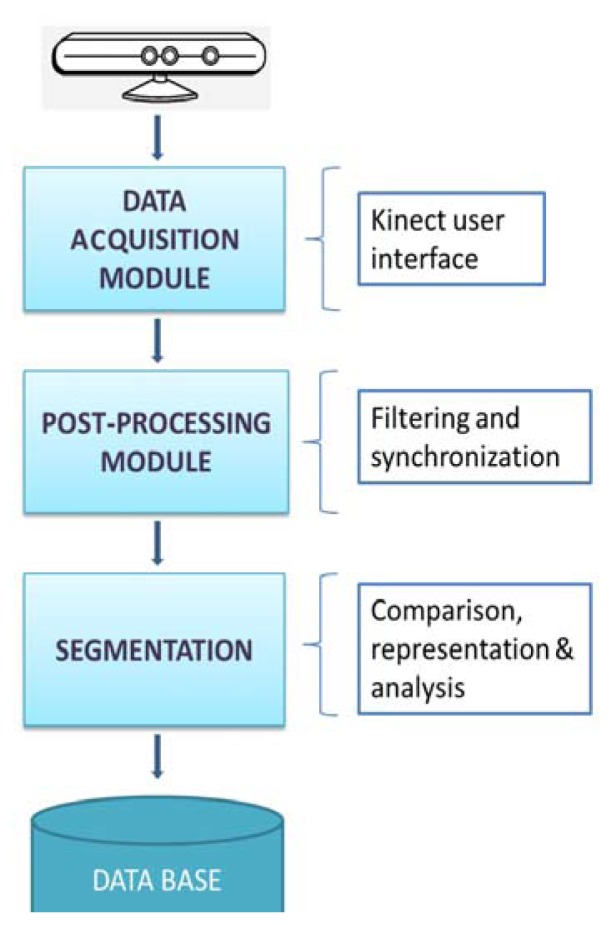
The figure shows the modular methodology used with the Kinect™ for data analysis. First, the data is acquired so as to launch the camera sensor using Kinect SDK^©^ functions and the interface developed, second, the data is filtered and synchronized, then the segmentation in epochs is carried out to represent and analyze the data. Finally, the information is stored.

According to the methodology to follow during the execution of the experiment, Figure 2 shows a block diagram representing the whole process designed since the data acquisition from the Kinect™ to the plot and analysis of the signal.

**Figure 3.**
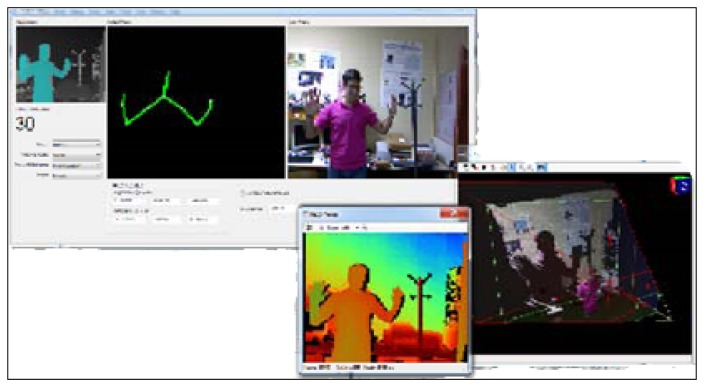
Kinect™ user interface.

First of all, a user interface (Figure 3) has been designed in order to interact with the Kinect™ and make the acquisition of the data easy.

This interface lets the user choose between different options related to the mode in which the Kinect™ records the information from the cameras. One of the advantages of selecting the mode is that the user can customize the video image. Instead of recording the whole body, the Kinect™ can record only the data from the upper half of the body. This feature is pertinent to the CogWatch system, which requires information obtained strictly from hand movements during ADL performance.

Besides the different images from the whole scenario, another functionality provided by the interface is the possibility of visualizing in real time the X, Y, Z positions of the hand (exactly, of a fixed point in the wrist). The interface, which has been programmed using C++ in Microsoft Visual Studio, has implemented a final option of saving all the position data in a file with a specific format selected by the user. The most common file formats used during the experiments are .xls and .csv.

Secondly, once all the data is saved, all the data is post-processed by loading the files saved before using Matlab in order to plot the signals and study if any filtering is needed. As shown in the following section, filtering has been focused on Butterworth. A Butterworth filter is applied to obtain maximum flatness in the band pass. For this article, it is applied in its digital version that is an IRR filter. The Butterworth filter provides the best Taylor Series approximation to the ideal low-pass filter response at analog frequencies f_r_ = 0 and f_r_ = ∞. In this case, no stability problems are found in the domain of the input data.

After filtering the signal, Kinect™ data is compared with the data obtained from Zebris, and used to determine whether the Kinect™ can recognize handmade movements, and if it is a suitable motion capture system for cognitive rehabilitation.

For comparison, an ultrasonic three-dimensional motion capture system was used as reference system. The Zebris [42,43] system features three sonic emitters, which send out packets of ultrasound, and receivers that can be placed on relevant body segments.

## 4. Experiments and Results

### 4.1. Data Collection

In order to test the suitability of the Kinect™ device as a motion capture system we collected data from a neurologically healthy 32 year-old female (control participant), and a 47 year-old female with apraxia (apraxic participant). Both participants were right handed. The apraxic participant had left-brain damage which resulted in hemiparesis of the dominant right hand. As such the apraxia patient had the use of the non-dominant left hand only.

**Figure 4.**
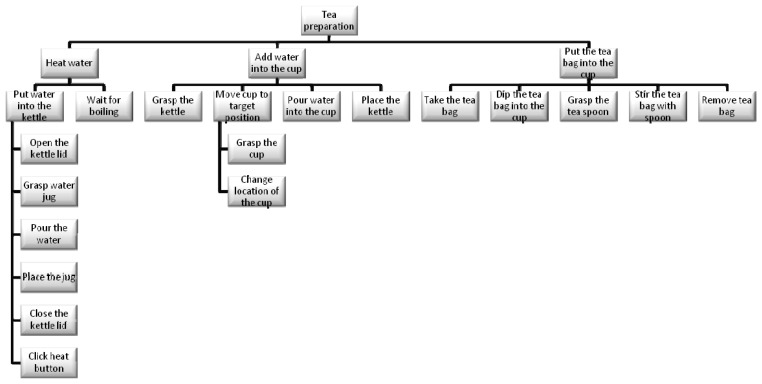
Task tree for tea preparation.

While the decomposition of an activity in component tasks is common across a range of disciplines, Human Factors (particularly in the UK) employ a methodology called Hierarchical Task Analysis (HTA) [44]. What is important in this approach is not simply the hierarchical decomposition but also the definition of ‘plans’. As in the psychological studies of Cooper and Shallice [33], hierarchy is typically described in terms of decomposition of a “goal” into “subgoals”, moving from a high-level objective to lower-level tasks. In this case and based on HTA, the whole task tree designed for the project and experiments regarding the task focused on tea preparation is shown in the next Figure 4.

However, in order to simplify the execution and make the experiment faster for the article, the task to be executed is reduced by following these instructions, which specify a particular sequence in which the task should be performed:

- Reach and grasp the cup.
- Transport the cup to target position.
- Reach and grasp the tea bag.
- Place the tea bag into the cup.
- Reach and grasp the kettle.
- Transport the kettle to position over cup.
- Pour the water from the kettle into the cup.
- Place the kettle back on table.
- Reach the tea bag.
- Stir the tea bag in the cup.

**Figure 5.**
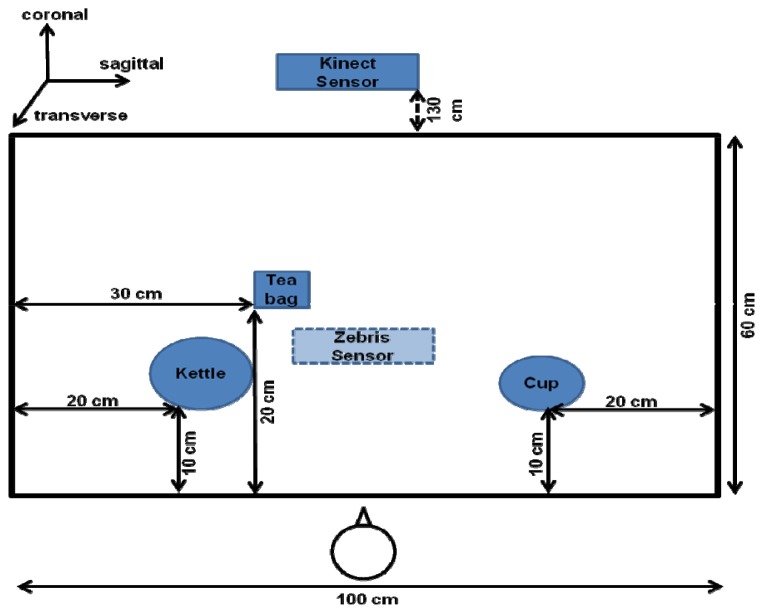
This figure shows the object layout for the experiment. The objects involved: a cup of tea, a kettle, a tea bag; the sensor Kinect™ and the Zebris system. The general Cartesian reference system is also represented.

Given that the apraxic participant was hemiparetic, the control participant was also required to use only the left hand to perform the task. The instructions emphasized that the task must be performed at a normal pace. The participants performed a total of 20 tea-making trials. The entire session took 30 minutes. The objects used in the experiment were arranged as shown in Figure 5.

The optimal distance to locate the Kinect™ sensor in front of the participants was 1.3 m. It was arranged so as to coincide with the participants body midline. By default, the way the Kinect™ detects and obtains data from the body of the user is divided into different steps: the first step is focused on the segmentation of the depth data per-pixel, body-part classification. In the second step, the system hypothesizes the body joints by finding global centroid of probability mass (local modes of density) through mean shift. In the last step, the hypothesized joints are mapped to the skeletal joints, and then fit to a skeleton by considering both temporal continuity and prior knowledge. Thus, the body is represented as a stick figure consisting of line segments linked by joints (e.g., head, neck, shoulders, and arms), with each joint represented by its 3D coordinates.

Kinematic data was collected by the Kinect™ (30 Hz sampling rate) and the Zebris systems (120 Hz sampling rate). The Zebris system [42,43] was used as reference system, and consisted of a sensor placed 1 m above the table, and a marker (1 cm diameter) which was fixed to the dorsum just proximal to the space between 1st and 2nd metacarpophalangeal joint of the left hand. The Zebris data was processed using WinData software 2.19.14 [42].

### 4.2. Data Analysis

**Figure 6.**
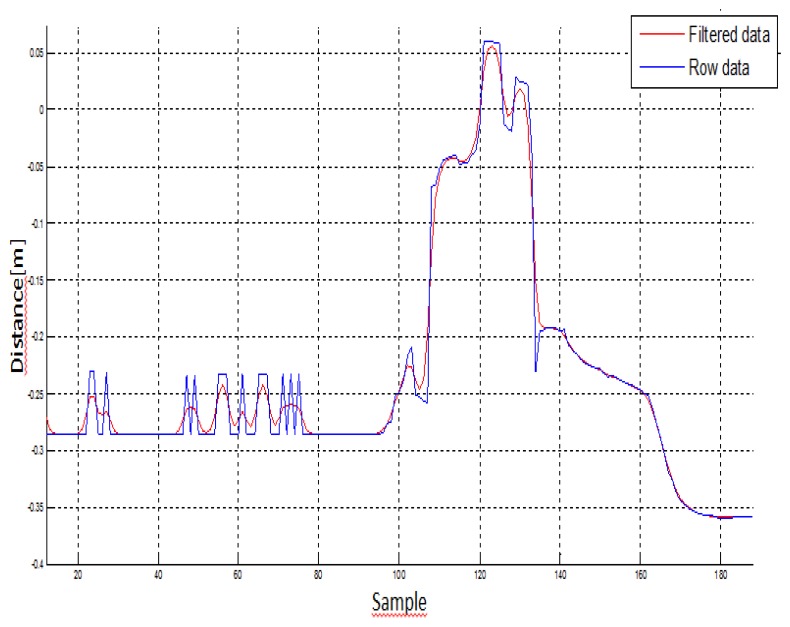
Result of filtering Kinect™ data with a Butterworth low pass filter.

In the first step of data analysis, the kinematic data was loaded in a custom written MatLab program (The MathWorks^®^, Version R2010a), and the 3D coordinates were low-pass filtered at a 5 Hz cut-off, using a first order Butterworth filter. The data was also filtered using an infinite response filter, which was selected because it reduced the amount of delay in the signal. It is known to be more computational efficiency than a finite response filter. This low-pass filter adequately smoothed the signal, rejected movement artefacts and high frequency phenomena (e.g., aliasing), and eliminated power line harmonic interferences. Figure 6 shows the signal before (blue signal) and after (red signal) filtering Kinect™ data:

In the next step, the Zebris data was then down-sampled to 30 Hz and then normalized by computing standardized z-scores using the mean and standard deviation of the vector for each axes (transverse, sagittal, coronal). This procedure allowed the comparison of the obtained data from the two motion capture systems to be done directly. The differences between the Kinect™ and Zebris data were quantified utilizing Mean Square Error (MSE) and cross-correlation measures separately for each axes. MSE was computed using the matlab mean squared normalized error performance (mse) function, which measures the expected squared distance between an estimator (Zebris) and the observations (Kinect™). A MSE of 0 means the estimator (Zebris) predicts observations (Kinect™) with perfect precision. Larger MSE values indicate that the observation values (Kinect™) differed from the estimator (Zebris).

Cross-correlations were computed by using the matlab corrcoef function, and that function was used for estimating the dependence of the values obtained from the two motion capture systems. The correlation coefficient ranges from −1 to 1. A value of 1 implies that a linear equation describes the relationship between the Zebris and Kinect™ perfectly. Values around 0 imply that there is no linear correlation between the variables. Negative values indicate an inverse relationship between the variables (*i.e.*, the values of one of the variables increase, the values of the second variable decrease).

In order to ascertain whether the comparison of obtained data from the Kinect™ and Zebris systems differed between the control participant and the apraxic participant, we conducted a 2 (individual: control participant, apraxic participant) × 3 (axes: transverse, sagittal, coronal) for each measure (MSE, cross-correlation) separately. The α level was set at 0.05 and as such p values less than 0.05 indicate statistical differences.

### 4.3. Results

**Figure 7.**
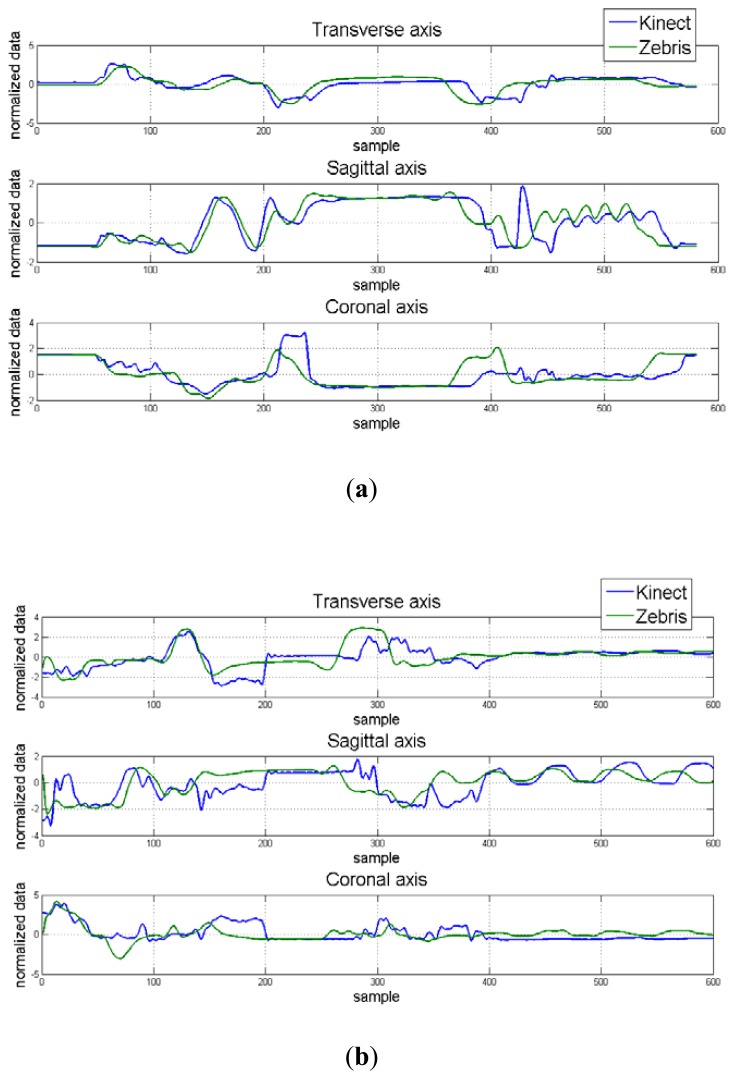
Comparison of position data for the Kinect (blue lines) and the Zebris system (green lines) from a single tea making task trial for the control (**a**) and the apraxic (**b**) participant.

Hand position for each dimension and correlation maps between axes for the control and apraxic participants are displayed in Figure 7. It was observed that the Kinect™ (blue signal) was able to adequate track 3D hand positions, and was similar to that collected by a Zebris marker-based ultrasonic motion capture system (green signal).

The average Mean Square Error (MSE) and cross-correlation values for each axis are displayed in Table 4. Considering MSE, the values for the control participant ranged from 0.938 to 1.143. The MSE values for the apraxic participant were slightly higher, ranging from 1.265 to 1.322. Statistical analysis indicated that MSE values for the control participant and apraxic patient were significantly different from one another, F(1,19) = 10.017, p = 0.005. This difference was primarily driven by the MSE values in the sagittal axes, which were lower for the control participant than the apraxic patient, F(1,19) = 4.478, p = 0.018.

These results indicate that the overall observation (Kinect™) and estimator values (Zebris) were similar to one another (*p* > 0.05) regardless of axes, and that the obtained data from the Kinect™ and Zebris motion capture systems were similar regardless of the neurological status of the participant for the transverse and coronal axes, but were significantly different from another in the sagittal axes.

On the other hand, cross-correlation analysis indicated that the relationship between the Kinect™ and Zebris systems ranged from 0.427 to 0.525 for the control participant. Based on the Cohen scale [45], these results indicate a medium correlation between motion capture systems for the transverse and sagittal axes, and a strong correlation between motion capture systems for the coronal axis. The cross-correlations for the apraxic patient ranged between 0.338 and 0.367, indicating a moderate correlation between the tested motion capture systems for all three axes. Statistical analysis indicated that the cross-correlations were similar between the control participant and the apraxic patient.

**Figure 8.**
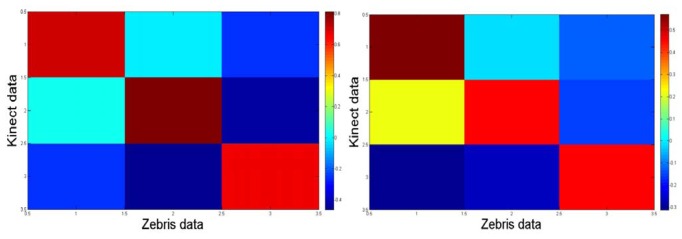
Correlation map between axes for the control participant (left panel) and the apraxic participant (right panel).

Notice from Figure 8 that the correlation map from the trials with the control participant is more symmetric than that one for the apraxic participant due to that the transformation between axes from the Zebris and the Kinect™ is more linear because of different set up configuration. The data was considered and processed consequently.

## 5. Conclusions

The purpose of the current experiment was to ascertain whether the Kinect™ device can be used as a motion capture system in a cognitive rehabilitative context. Overall, the results indicated a moderate to strong correlation between signals in the control participant, and moderate correlations between signals in the apraxic participant. Furthermore, although the sagittal axes MSE values differed between the control participant and apraxic one, the transverse and coronal axes MSE values were similar for both participants. Taken together, the research presented indicates that the Kinect™ device is able to adequately track hand movement during ADL, regardless of the neurological status of the individual.

These findings are indeed promising given that the sampling frame of the Kinect™ device is lower than the Zebris system (30 Hz compared to 120 Hz), the position of the hand is determined using RGB camera and depth camera, and that the Kinect™ sensor costs significantly less than its marker-based counterparts.

Moreover, one of the main advantages of the Kinect™ unit is its continuous development. It expands the possibilities for innovation with features like Near Mode, which enables the depth camera to see objects as close as 40 centimetres in front of the sensor. In addition, up to four Kinect™ sensors can now be plugged into the same computer and do not need any calibration (if used on Windows). Such possibility is worth mentioning since it can improve and increase the accuracy of tracking and future possible recognition of movements from the kind of patients treated as presented in the article.

Although the applications developed in the CogWatch project will be initially used by clinicians, Kinect™ could also be a useful tool for home rehabilitation as proven in the results. It is actually the main objective to be reached at the final stages of this project.

As future work, more detailed experiments will involve multiple ADL tasks (e.g., making toast, putting on a shirt, *etc.*) and various AADS patient populations (e.g., aphasic). Overall, while more researches could be done in the capabilities and limitations of the device in physical therapy, this work has found out that Kinect™ has great potentials to apply in stroke rehabilitation as a tool for both clinicians and stroke survivors.

## Figures and Tables

**Table 1.** This table shows the main projects focused on different types of post-stroke rehabilitation. The projects are classified as follows; Passive, when the system does not supply response, Active assisted, when the system guides the movements of the patient and Active resisted, when the aim of the system is to fit the patient training movements against a resistance.

**Passive**	**Active Assisted**	**Active Resisted**	
MIME System			**Past Projects**
ARM Guide			
MIT Manus		GENTLE/S	
Rigid Exoskeleton/Rigid Link Manipulation			**More Recent Projects**
Soft Natural Anatomic Structures			
Active Soft Orthotic System			
Electrical Stimulations			

**Table 2.** This table summarizes some of the most probably common effects of apraxia in patients. These effects can appear combined and give an idea of the difficulties that patients have to deal with.

**Type of Apraxia**	**Deficit Involved**

Ideo motor	Hard moves using tools or making gestures
Kinematic	Dexterity, manipulation
Ideational	Secuential tasks with multiple objects
Conceptual	Knowledge of tools and their use

**Table 3.** Kinect™ principal features for capturing image video and motion.

**Sensor Item**	**Specification Range**

Viewing angle	43° vertical by 57° horizontal field of view
Mechanized vertical tilt	±28°
Frame rate (depth and color stream)	30 frames per second (FPS)
Resolution, depth stream	QVGA (320 × 240)
Resolution, color stream	VGA (640 × 480)

**Table 4.** Mean Square Error (MSE) and cross-correlation values for the transverse, sagittal, and coronal axes.

	**Mean Square Error (MSE)**			**Cross-Correlation**		

	**Transverse**	**Sagittal**	**Coronal**	**Transverse**	**Sagittal**	**Coronal**
**Control**	1.143	0.938	1.074	0.427	0.489	0.525
**Apraxic**	1.265	1.322	1.292	0.367	0.338	0.353
